# Predictors for early introduction of solid food among Danish mothers and infants: an observational study

**DOI:** 10.1186/1471-2431-14-243

**Published:** 2014-10-01

**Authors:** Hanne Kronborg, Else Foverskov, Michael Væth

**Affiliations:** Department of Public Health, Aarhus University, Bartholins Allé 2, 8000 Aarhus C, Denmark; Section for Nursing, Department of Public Health, Aarhus University, Hoegh-Guldbergs Gade 6A, 8000 Aarhus C, Denmark; Section for Biostatistics, Department of Public Health, Aarhus University, Bartholins Allé 2, 8000 Aarhus C, Denmark; Department of Social Policy, London School of Economics and Political Science, Houghton Street, London, WC2A 2AE UK

**Keywords:** Infant feeding practices, Breast-feeding, Introduction of complementary feeding, Solid food, Infant temperament, Risk factors

## Abstract

**Background:**

Early introduction of complementary feeding may interfere with breastfeeding and the infant’s self-controlled appetite resulting in increased growth. The aim of the present study was to investigate predictors for early introduction of solid food.

**Methods:**

In an observational study Danish mothers filled in a self-administered questionnaire approximately six months after birth. The questionnaire included questions about factors related to the infant, the mother, attachment and feeding known to influence time for introduction of solid food. The study population consisted of 4503 infants. Data were analysed using ordered logistic regression models. Outcome variable was time for introduction to solid food.

**Results:**

Almost all of the included infants 4386 (97%) initiated breastfeeding. At weeks 16, 17–25, 25+, 330 infants (7%); 2923 (65%); and 1250 (28%), respectively had been introduced to solid food. Full breastfeeding at five weeks was the most influential predictor for later introduction of solid food (OR = 2.52 CI: 1.93-3.28). Among infant factors male gender, increased gestational age at birth, and higher birth weight were found to be statistically significant predictors. Among maternal factors, lower maternal age, higher BMI, and being primipara were significant predictors, and among attachment factors mother’s reported perception of the infant as being temperamental, and not recognising early infant cues of hunger were significant predictors for earlier introduction of solid food. Supplementary analyses of interactions between the predictors showed that the association of maternal perceived infant temperament on early introduction was restricted to primiparae, that the mother’s pre-pregnancy BMI had no impact if the infant was fully breastfed at week five, and that birth weight was only associated if the mother had reported early uncertainty in recognising infant’s cues of hunger.

**Conclusions:**

Breastfeeding was the single most powerful indicator for preventing early introduction to solid food. Modifiable predictors pointed to the importance of supporting breastfeeding and educating primipara and mothers with low birth weight infants to be able to read and respond to their infants’ cues to prevent early introduction to solid food.

## Background

It is recommended by the WHO not to introduce complementary food to infants below six months of age [1, 2] as a prolonged period of exclusive breastfeeding prevents a number of infectious diseases and is associated with a slower weight gain during the second half year of life [1]. Moreover, a prolonged period of exclusive breastfeeding seems to have a positive impact on cognitive development, and a protective effect concerning development of chronic diseases such as type 1 diabetes [3].

Early introduction of complementary food tends to interfere with breastfeeding and increase growth leading to an increased risk of developing child obesity [4–9]. It is unknown whether it is the preventive effect of breastfeeding and the dose of human milk or whether early complementary feeding is critical in increasing infant weight gain [3–10]. Another factor connected with early introduction of complementary food is the potential risk of increased parental control with energy intake, which may interfere with the infant’s self-controlled appetite [11].

Complementary feeding can roughly be divided into infant formula food/bottle feeding and solid food/spoon food. Early introduction of formula is complementary to early cessation of exclusive breastfeeding [12, 13]. Previous research has focused on reasons for stopping exclusive breastfeeding and thereby early introduction of formula food. Factors influencing early introduction of solid food have only been sparsely investigated. Although the time for introduction of solid food has been delayed during the last decade, approximately 10% of mothers in Scandinavia [14], 30% in the UK [15], and 21% in the US still introduce the infant to solid food before four months of age [16, 17].

Known reasons for early introduction of solid food are related to both the mother and the infant. Maternal characteristics for early introduction have previously been linked to socio-demographic and psycho-social determinants of behavior concerning attachment. Among socio-demographic determinants low level of education [9, 15], smoking [13], lower age [13, 15], and increased maternal pre-pregnant body mass index (BMI) tend to determine the family’s feeding practice and to be associated with early introduction of solid food [4, 5, 10, 18]. Moreover, low income groups seem to take advice from family members rather than complying with health recommendations [19]. Attachment factors such as the mother’s perception of infant’s signs of hunger and satiety, beliefs in solid food to address concerns about feeding problems or to extend sleep [13, 19, 20], the mother’s perception of infant’s temperament [21, 22], and new parents’ parental confidence [18] have earlier been associated with timing of introduction of solid food. Among infant factors infant boys and infants with high birth weight and early rapid weight gain are more likely to have been introduced to solid food during the first four months [5].

The known associations are presented in different studies using different study designs and no single study has so far included all factors. In Denmark, nearly all mothers start breastfeeding after birth. This provides a unique basis for investigating feeding practices and transition from breastfeeding to complementary feeding during the infant’s first six months of life. The aim of the present study was to investigate predictors for early introduction of solid food, in particular the association between socio-demographic, attachment and infant factors and feeding practices and the timing of introducing solid food.

## Methods

### Design, setting, participants

A cross-sectional study design was used to collect data among women who had given birth six months ago. The study took place in the western part of Denmark and included 19 municipalities in both urban and rural areas with an annual birth rate of approximately 15000 births. In Denmark, almost 99% of all deliveries take place in hospitals; in the following months health visitors offer support in relation to the infant’s emotional, nutritional and developmental needs.

In Denmark all citizens are assigned a unique civil registration number at birth provided by the Civil Registration System. This number was used to identify newborns and their mothers in the study region. Women were recruited during a five-month period from 1 April to 31 June and from 1 August to 31 October 2008, leaving the holiday season in July without data collection. All women who lived in the study area and had a newborn registered on their address in the periods were invited to participate in the study.

### Data collection and questionnaire

Data were collected from eligible mothers received an anonymous, self-administered questionnaire approximately six months after birth together with a pre-paid return envelope. The questionnaire included socio-demographic questions, questions related to maternal perception of early attachment, breastfeeding and infant temperament, questions about infant growth and well-being, and questions about the service received from the health care system.

The questionnaire consisted mainly of questions used in earlier studies [23, 24] and had in that connection been face and content validated. New questions were developed for this study to collect information on complementary feeding. The questionnaire was subsequently reviewed by two experts and pre-tested for comprehension and acceptability in two rounds by 12 mothers who represented different age, parity as well as social and ethnic backgrounds.

### Variables/measures

**Figure 1:**
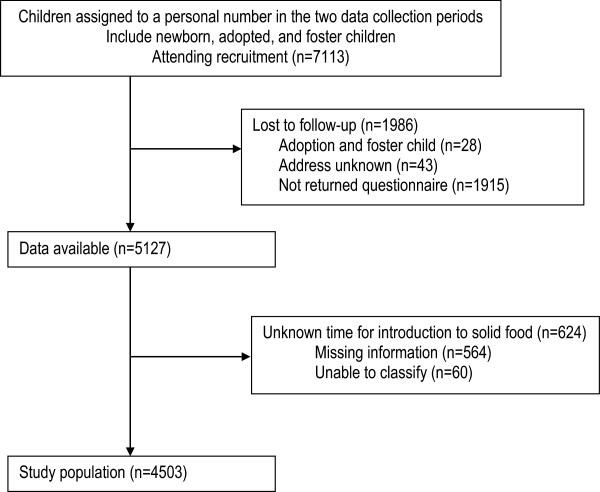
**Flow profile and exclusion criteria for selection of study population.**

All variables were collected from the self-reported questionnaire.The outcome variable was time for introduction of solid food. To measure time for introduction of solid food mothers were asked: how many months and weeks was your child when you started spoon feeding with mash or porridge? The answers were categorised into three time periods: 5–16 weeks, 17–25 weeks, more than 25 weeks (week 25+). Cross-checking was conducted by comparing mothers’ reported time of introduction to solid food with their reported duration of full breastfeeding. The outcome variable was set to missing for 60 cases with conflicting responses (Figure 1).

Study variables included variables related to both mother and infant. Maternal factors included: socio-demographic, attachment, and feeding factors. Socio-demographic factors included questions to ethnicity, age, educational level, cohabitation status, smoking status, pre-pregnancy body mass index (BMI), self-reported ethnicity and parity. Attachment factors were measured by asking the mother about early perception of recognition of infant cues and later perception of infant temperament. Early attachment factors included the mother’s perception of within the first five weeks postpartum being able to be calm and comfort her baby, understand the infant’s needs, and recognize the cues of hunger; response categories were, “always”, “usually”, “seldom”, and “never”. Answers were categorised in “always” and “not always” (latter category including “usually”, “seldom”, and “never”). Mother’s later perception of infant temperament was measured by asking: how is your child’s temperament now generally speaking?; response categories were, “very fiery temper”, “as average, normal”, “a docile temperament”. Answers were categorised in “very temperamental” and “not temperamental” (latter category including “as average, normal” and “a docile temperament”). Feeding factors included questions related to duration of full and partial breastfeeding, and time for introduction of formula. Duration of full and partial breastfeeding was measured using a slight modification of indicators for assessing breastfeeding practices (21) by asking the mother: How many months and weeks did you breastfeed your baby without giving any supplement at all? (Full breastfeeding was converted to weeks); how many months and weeks was your baby when you fed s/he something other than your milk from a bottle or a cup more than once a week? (Partial breastfeeding was converted to weeks). How many months and weeks was your baby when you stopped breastfeeding? (No breastfeeding was converted to weeks). To measure the time of introduction of bottle feeding, mothers were asked: how many millilitres of formula did your baby receive during 24 hours when s/he was one month, two months etc. (Formula milk feeding). Child factors included: gender (male/female), gestational age at birth (weeks) and birth weight (kg).

### Statistical analysis

Initially, the association between the time of introduction of solid food and each of the potential predictor variables among maternal socio-demographic-, attachment-, feeding-, and infant factors were assessed separately by chi-square tests for categorical characteristics and unadjusted ordered logistic regressions for continuous characteristics.

Next, a multiple ordered logistic regression model was used to simultaneously assess the risk factors identified as statistically significant in bivariate analyses. This analysis generalises a (binary) logistic regression analysis of outcome variables with more than two ordered categories. The results were presented as adjusted odds ratios for timing of introduction of solid food with 95% confidence intervals. For a categorical variable, an odds ratio larger than 1 shows that the particular category is associated with a delayed introduction of solid food relative to the reference category. For a continuous variable, the odds ratio gives increased odds associated with a one-unit increase of the predictor. All continuous variables were centered before entrance into the logistic regression model: Mother’s age at 30 years, mother’s BMI at 25, infant birth weight at 3.5 kilogram and gestational age at birth at 39 weeks. For categorical variables reference categories were chosen according to expected unfavourable prognosis. Only the variables that retained statistical significance in the final model are presented in Table 1.

**Table 1:** **Infant, maternal, attachment and feeding characteristics of 4,503 mother-child pairs according to time for introduction to solid food**

		Introduction to solid food						
		Week 5–16 (N 330)		Week 17–25 (N 2923)		Week 25+ ( N 1250)		
Variable	Values	N	%	N	%	N	%	***p value***
**Infant factors**								
Gestational age at birth in weeks	Mean (SD)	314	39.6 (1.9)	2811	39.5 (1.9)	1191	39.1 (2.5)	< 0.001
Birth weight in kilogram	Mean (SD)	323	3.6 (0.6)	2895	3.5 (0.6)	1234	3.4 (0.7)	< 0.001
Sex	Girl	148	44.85	1389	47.52	661	52.88	0.002
	Boy	182	55.15	1534	52.48	589	47.12	
**Maternal factors**								
Age in years	Mean (SD)	326	29.7 (5.1)	2890	30.8 (4.4)	1233	31.9 (4.5)	< 0.001
Body mass index	Mean (SD)	318	25.8 (6.3)	2843	24.3 (5.1)	1209	24.0 (4.7)	< 0.001
Level of education	Non or short	187	58.62	1290	45.23	462	37.84	< 0.001
	Intermediate	110	34.48	1135	39.80	507	41.52	
	Long	22	6.90	427	14.97	252	20.64	
Smoking	Yes	64	19.57	309	10.68	87	7.04	< 0.001
	No	263	80.43	2583	89.32	1149	92.96	
Has a spouse or partner	Yes	312	95.41	2796	96.68	1196	96.92	0.400
	No	15	4.59	96	3.32	38	3.08	
Self-reported ethnicity	Danish	307	94.17	2712	93.71	1143	92.48	0.289
	Other than Danish	19	5.83	182	6.29	93	7.52	
Parity	Primipara	159	48.77	1335	45.94	491	39.53	< 0.001
	Multipara	167	51.23	1571	54.06	751	60.47	
**Attachment factors**								
Understand infant needs weeks 0-5	Always	46	14.15	395	13.62	226	18.36	< 0.001
	Not always	279	85.85	2505	86.38	1005	81.64	
Calm and comfort the infant weeks 0-5	Always	149	45.71	1283	44.18	608	49.55	0.007
	Not always	177	54.29	1621	55.82	619	50.45	
Recognise cues of hunger weeks 0-5	Always	136	41.85	1108	38.14	559	45.37	< 0.001
	Not always	189	58.15	1797	61.86	673	54.63	
Perceived infant temperament	Very temperamental	58	17.79	373	12.87	118	9.51	< 0.001
	Not temperamental	268	82.21	2525	87.13	1123	90.49	
**Feeding factors**								
Breastfed or formula-fed at week 5	Only formula-fed	57	17.38	252	8.66	40	3.22	< 0.001
	Formula and breastfed	59	17.99	469	16.11	173	13.91	
	Only breastfed	212	64.63	2190	75.23	1031	82.88	

Note: Missing values excluded, p-values are from Chi-square tests for categorical characteristics and from unadjusted ordered logistic regression for continuous characteristics. Figures are numbers and percentage unless stated otherwise.

Finally, all possible interactions between variables from attachment and feeding factors, respectively and variables from infant and maternal factors were investigated and statistically significant interactions were included in the final model. A Brant test of the proportional odds assumption was carried out on the final model [25].

The level of significance was chosen as 0.05. Stata version 12 was used for all statistical analyses [26].

### Ethics

The study was approved by The Central Denmark Region Committee of Biomedical and Research Ethics (Jr. no. 1-16-02-1-08/068) and the Danish Data Protection Agency (Jr. no. 2007-58-0010). Written information of the study was provided to the women before enrollment.

## Results

A total of 7113 newborns were registered in the study region during the study periods. Of these, data were available for 5127 (72%). Reasons for not enrolling in the study were: mother did not return the questionnaire, address unknown, or foster infant or adoption. The study population consisted of 4503 (63%) infants after exclusion of 624 (9%) infants with incomplete information on introduction to solid food. The excluded mothers and infants showed no statistically significant difference with respect to mother’s age, educational level, gestational age at birth, or birth weight compared to included mothers and infants. A total of 82% of the questionnaires were returned within the first 32 weeks; 97% within 40 weeks postpartum.

Almost all the included infants 4386 (97%) initiated breastfeeding after birth. At week five postpartum, 349 (8%) infants were formula fed; 701 (16%) infants were partially breastfed, and 3433 (76%) were still fully breastfed. At week 16, 330 infants (7%) had been introduced to solid food; 2923 (65%) were introduced to solid food between week 17–25, and 1250 (28%) later than week 25.

Table 1 shows the characteristics of infants and mothers stratified according to time of introduction of solid food. A significant difference was noted between the three groups of infants introduced to solid food in weeks 5–16, in weeks 17–25, or in week 25+, with respect to gender of the child (p = 0.002), lower gestational age at birth and birth weight (p < 0.001). Mothers introducing their infants to solid food later were characterized by being multipara (p < 0.001), significantly older (p < 0.01), had a lower pre-pregnancy BMI (p < 0.001), a higher educational level (p < 0.001), and were non-smokers (p < 0.001). Moreover, delayed introduction to solid food was associated with mothers not perceiving their infants as temperamental (p < 0.001), and mothers always being able to understand their infant in the first five weeks in relation to: comfort the infant (p < 0.001), understand the infant’s needs (p = 0.007), and recognize cues of hunger (p < 0.001).

Table 2 shows the results of the multiple ordered logistic regression analysis. Model 1 includes the statistically significant factors from the single-factor analyses without including interactions. Model 2 includes also the statistically significant interactions between factors included in Model 1. The odds ratios associated with variables not entering an interaction term were very similar in the two models. Brant’s test of the proportional odds assumption showed no overall violation of the assumption for any of the models (Model 1: p = 0.116; Model 2: p = 0.184). Girls were introduced to solid food later than boys and increased gestational age at birth and high birth weight were associated with earlier introduction of solid food. Statistically significant maternal factors included the age of the mother; the odds for introducing solid food after week 25 increased by 5% (OR = 1.05 CI:1.04-1.07) for every year the mother was older. A higher level of education, being a non-smoker or multipara also influenced the odds for introducing solid food later. Among the attachment factors, mothers’ reported perception of infant temperament as being average and ability to being able early to recognise the infants’ cues of hunger were significantly associated with later introduction of solid food. Among feeding factors, full breastfeeding at week five more than doubled the likelihood for being introduced to solid food at a later age (OR = 2.52 CI: 1.93-3.28) (Table 2).

**Table 2:** **Associations between introduction to solid food (weeks 5–16, weeks 17–25, weeks 25+) and infant, maternal, attachment and feeding characteristics estimated by ordered logistic regression (N = 4066)**

		Model 1		Model 2	
Characteristics	Value	OR	95% CI	OR	95% CI
**Infant factors**					
Gestational age at birth	Weeks	0.95*	0.92:0.99	0.96*	0.92:1.00
Weight at birth	Kilograms	0.78**	0.68:0.90	0.70***	0.59:0.83
Sex^a^	Girl	1.21**	1.06:1.38	1.20**	1.05:1.37
**Maternal factors**					
Age	Years	1.06***	1.04:1.07	1.05***	1.04:1.07
BMI	Mass(kg) / Height(m)^2^	0.99*	0.97:1.00	0.96	0.93:1.00
Education^b^	Intermediate education	1.18*	1.02:1.37	1.19*	1.03:1.38
	Long education	1.52***	1.25:1.84	1.52***	1.25:1.85
Smoking^c^	No	1.52***	1.21:1.91	1.52***	1.21:1.92
Parity^d^	Multipara	1.15	0.99:1.33	1.73**	1.17:2.57
**Attachment factors**					
Recognise cues of hunger week 0-5^e^	Always	1.27***	1.11:1.45	1.27**	1.11:1.45
Perceived infant temperament^f^	Not temperamental	1.45***	1.18:1.78	1.77***	1.35:2.33
**Feeding factors**					
Breastfed or formula-fed at week 5^g^	Formula and breastfed	1.75***	1.30:2.35	1.72***	1.27:2.33
	Only breastfed	2.51***	1.94:3.26	2.52***	1.93:3.28
**Interactions**					
BMI x feeding in week 5^h^	BMI for formula and breastfed			0.99	0.94:1.03
	BMI for only breastfed			1.03	0.99:1.08
Parity x perceived temperament^i^	Multipara and not temperamental			0.63*	0.42:0.95
Birth weight x recognise cues^j^	Kilograms and always			1.29*	1.03:1.62

Note: Model 1 included all main effects of factors that were statistically significant in single-factor analyses. Model 2 included also statistically significant interactions between these factors.

Associations are expressed as adjusted odds ratios. Missing values excluded. Wald test of the interaction between maternal BMI and feeding in week 5 : p = 0,011.

Reference categories: ^a^Boy, ^b^None or short education, ^c^Yes, ^d^Primipara, ^e^Not always, ^f^Very temperamental, ^g^Only formula-fed, ^h^BMI and Only formulafed, ^i^Primipara and Very temperamental, ^j^Kilograms and Not always.

*p < 0.05, **p < 0.01, ***p < 0.001.

The details of the interactions are further described in Table 3 where odds ratios and 95% confidence intervals were calculated for a range of values of the variables entering the interaction terms. The interactions included in Model 2 showed that the importance of perceived infant temperament was restricted to primiparae women, that the mother’s pre-pregnancy BMI was unimportant if the infant was only breastfed at week five and that the birth weight was particularly important if the mother had reported uncertainty about recognizing the infant’s cues of hunger in the first five weeks.

**Table 3:** **Odds ratios and 95% confidence intervals for selected values of the variables entering the interaction terms in Model 2**

	OR	95% CI
**Interaction: Parity and perceived infant temperament**		
Primipara		
Very temperamental	1.00	
Not temperamental	1.77	[1.35:2.33]
Multipara		
Very temperamental	1.00	
Not temperamental	1.12	[0.82:1.52]
**Interaction: Formula-fed or breastfed at week 5 and maternal BMI**		
Only formula-fed		
BMI 20	1.20	[0.99:1.45]
BMI 25	1.00	
BMI 30	0.84	[0.69:1.01]
BMI 35	0.70	[0.48:1.03]
Formula and breastfed		
BMI 20	1.28	[1.11:1.50]
BMI 25	1.00	
BMI 30	0.78	[0.67:0.90]
BMI 35	0.61	[0.45:0.82)
Only breastfed		
BMI 20	1.01	[0.94:1.09)
BMI 25	1.00	
BMI 30	0.99	[0.91.1.07]
BMI 35	0.98	[0.84:1.14]
**Interaction: Recognise cues of hunger weeks 0–5 and birth weight (BW)**		
Not always recognise cues of hunger		
BW 3.0	1.20	[1.10:1.30]
BW 3.5	1.00	
BW 4.0	0.84	[0.77:0.91]
Always recognise cues of hunger		
BW 3.0	1.05	[0.95:1.16]
BW 3.5	1.00	
BW 4.0	0.95	[0.86:1.05]

Note: The values of body mass index (BMI 20, 25, 30, 35) and birth weight (BW 3.0, 3.5, 4.0) are the values for which the odds ratio is calculated.

## Discussion

The majority of Danish infants were introduced to solid food between four and six months after birth; only a small proportion was introduced before four months and approximately one third after six months of age. Infant characteristics such as female gender, lower gestational age at birth, lower birth weight and maternal characteristics including multipara, older age, higher level of education and non-smoking status showed to be protective against early introduction of solid food. The most influential factor was being fully breastfed at five weeks which doubled the chance for a delayed introduction of solid food. The interaction analysis showed that the association between early introduction of solid food and the mother’s perception of the infant being very temperamental was primarily found among primiparae. The association with birth weight was particularly strong if the mother had difficulty recognising the infant’s cues of hunger in the first five weeks. Moreover, high maternal pre-pregnancy BMI did not associate with the time for introduction of solid food as long as the infant was fully breastfed at five weeks.

The importance of breastfeeding at five weeks in the present study agrees with the findings of Scott et al. [13] who also found that this was the most important indicator for preventing early introduction to solid food. Among a number of positive health outcomes, breastfeeding also benefits a healthier growth pattern [3], whereas formula feeding may result in weight gain over a longer period [27]. Compared to breastfed infants, bottle fed infants generally have a higher protein intake and a tendency to drink more milk in the second half year of life after introduction of solid food [28, 29]. This may be explained by the absence of the breastfeeding regulation of appetite control or relate to the bottle-feeding mother behaving more restrictive and less responsive to the infant’s needs [11, 30]. There may also be a biological explanation like the absence of the hormone leptin in formula milk; in breast milk this hormone seems to have a regulating capacity to reduce appetite and increase metabolism [31]. Huh et al. [6] found that the timing of solid food was not associated with obesity at the age of three years among breastfed infants, whereas among formula feed infants introduction of solid food before four months was associated with a six-fold increase in the risk of obesity at the age of three years. Our results suggest that breastfeeding may be an independent predictor for an appropriate transition phase to solid food confirming that guidance in infant-feeding practice to new parents should start with breastfeeding support and focus on continuing breastfeeding also after introduction of solid food.

This study in accordance with other studies showed that mother’s younger age [13, 18], lower level of education [18], and smoking status [13] were associated with earlier introduction of solid food. In Western societies these socio-demographic factors seem to be associated with a reduced likelihood of following health recommendations, in this case complying with WHO recommendations on infant feeding [32]. This type of socio-demographic information is typically available before the health professionals meet the family and thereby point to some early identifiable risk factors for earlier introduction of solid food. These socio-demographic factors are, however, not easily changed, and attachment factors like the mother’s perception of infant’s hunger and infant temperament are therefore much more useful as predictors for health professionals working with early infant-feeding as they point to factors that are potentially modifiable.

We found that time for introduction of solid food was related to the mother’s perception of infant hunger, not her perception of infant satiety (results not shown). This is in accordance with the findings of Gross et al. [33] who found it easier for mothers to perceive infant satiety than cues of hunger, which for many mothers was connected to infant crying and associated with a more pressuring feeding style. Other researchers have found that mothers introduced solid food earlier if their infant seemed hungry [34], that mothers less able to respond to infant cues were more likely to introduce solid food earlier [18], and that mothers’ concerns about their babies getting enough to eat influenced the time for introduction of solid food [32]. The finding that the impact of the mother’s perception of infant cues on earlier introduction to solid food was related to a lower birth weight of the baby corresponds to earlier findings by Boyington et al. [35]; they found that infants perceived as small were introduced to solid foods earlier. The present findings contribute to the existing modest knowledge in this research area by identifying that guidance to mothers in reading their infants’ cues on feeding is especially important if the infant has a low birth weight. Otherwise, introduction of solid food may depend on the mother’s uncertainty rather than by the infant’s developmental readiness.

Only among primiparae the perception of infant temperament was associated with the time for introduction of solid food in the present study. The relation between maternal perception of infant temperament and rapid infant weight gain was demonstrated by Carey [36] already in 1988. Since then the correlation between infant temperamental characteristics perceived by the mother and infant feeding transition has been demonstrated by Nigel et al. [37] who found an association between mothers’ perception of difficult infant temperament at six months and shorter breastfeeding duration among Norwegian mothers. Wasser et al. [21] found an association between perceived infant temperament and earlier introduction to solid food among lower income US mothers. Our new findings that perceived infant temperament especially relate to time for introduction of solid food among primiparae may reflect a greater extent of anxiety and uncertainty connected to early infant care among primiparae compared to multipara [38]. The infant’s ability to regulate calorie intake supports a responsive feeding style in the transition phase from milk to solid food to sustain this self-regulation [39]. The risk connected with the perception of the infant being very temperamental may result in less awareness of the infant’s cues [22] and a more pressing controlling feeding style in the transition phase [40] leading to a care-giver oriented strategy more than an infant oriented strategy [41]. These findings support that learning to understand infant cues is especially important among primiparae to promote a positive mother-infant interaction [42]. Recent research points to how a gentle introduction with repetition of a variety of flavours facilitates the infants’ acceptance of different kinds of food also calls for educating primiparous mothers to be sensitive and responsive to infant cues [43].

We found no association between a higher maternal pre-pregnancy BMI and an earlier introduction to solid food if the infant was fully breastfed past five weeks. A high maternal pre-pregnancy BMI has so far primarily been shown to have a negative association with duration of breastfeeding [44–47]. Moreover, this association seems to be modified by parity and positive previous breastfeeding experience [48]. Unfortunately neither Scott et al. [13] nor Tatone-Tokuda et al. [18] included maternal BMI in their studies of predictors for early introduction of solid food. Our findings are in line with Baker et al. [10] who have found an interaction between higher maternal BMI, shorter duration of breastfeeding and earlier introduction of complementary food among Danish women [10]. The association between mother’s BMI and time for introduction to solid food depends apparently on her breastfeeding status. However, further research is needed to clarify the association between breastfeeding, maternal BMI and time for introduction of solid food.

The cross sectional design in which all data were collected at the same time is a limitation and thus, we cannot draw any causal conclusions from this study. Another limitation is the use of self-reported data related to nutrition and attachment. The study benefits from a large sample size with a response rate of 72% but we had to reduce the included study population in the present study to 63% of those eligible because of incomplete information on introduction to solid food. We know the excluded 9% of the mothers did not differ from included mothers concerning socio-demographic variables but otherwise we have no knowledge of the behaviour of the non-responding mothers. Data were collected when infants were six months and close to the time of introduction of complementary food. This may have reduced the risk of recall bias on the outcome factor, time for introduction of solid food. Breastfeeding duration is usually well recalled by mothers [49]. According to attachment factors asking about early interpretation of infant cues may have caused recall bias because mothers who have problems when infants are six months are more disposed to look for problems earlier on.

In the analysis the inclusion of infant, maternal, attachment and feeding factors which have until now been shown to be important to time of introduction of solid food and the follow-up for interactions between factor in continuation of the multivariate analysis increase the reliability of the present results. The outcome factor was cut-off at 16 weeks (5–16 weeks), 17–25 weeks and more than 25 weeks because the lower limit of Danish recommendations for introduction of solid food is 16 weeks (four months) with an acceptable limit between 17–25 weeks (four-five months), and a preferable limit after 25 weeks (six months) [50]. According to feeding factors, we included maternal feeding status at five weeks. This gave us an opportunity to distinguish between and adjust for full and partial breastfeeding or formula feeding in the analysis which has earlier been shown to be related to the time for introduction of solid food [4]. The cut-off at five weeks was chosen because nearly all mothers initiate breastfeeding after giving birth in Denmark; the first weeks are a learning phase of establishing or giving up breastfeeding. Early breastfeeding problems were not included in the analysis as they were considered predictors for early introduction of formula [12] more than predictors for solid food.

## Conclusions

The majority of Danish infants were introduced to solid food between four and six months and only a small proportion was introduced before four months. A number of non-modifiable infant and mother characteristics such as female gender, lower gestational age at birth and birth weight, being multipara, older age, and having a higher level of education showed to be protective against early introduction of solid food. The most influential factor was being fully breastfed at five weeks which more than doubled the likelihood of a delayed introduction to solid food. Among attachment factors, especially mothers’ perceived infant temperament among primiparae and having difficulty recognising infant cues of hunger in infants with low birth weight played a role in the time for introduction of solid food. Moreover, a high maternal pre-pregnancy BMI showed a significant impact on the time for introduction of solid food among mothers who did not fully breastfeed at five weeks. These more modifiable factors pointed to the importance of supporting breastfeeding and educating especially primipara and mothers with small infants to be able to read and respond to infant cues to prevent early introduction to solid food.
